# miR-324-5p is up regulated in end-stage osteoarthritis and regulates Indian Hedgehog signalling by differing mechanisms in human and mouse^☆^

**DOI:** 10.1016/j.matbio.2018.08.009

**Published:** 2019-04

**Authors:** Steven Woods, Matt J. Barter, Hannah R. Elliott, Catherine M. McGillivray, Mark A. Birch, Ian M. Clark, David A. Young

**Affiliations:** a Division of Cell Matrix Biology and Regenerative Medicine, Faculty of Biology, Medicine and Health, University of Manchester, Michael Smith Building, Oxford Road, Manchester M13 9PT, UK; b Skeletal Research Group, Institute of Genetic Medicine, Central Parkway, Newcastle University, Newcastle upon Tyne NE1 3BZ, UK; c MRC Integrative Epidemiology Unit at the University of Bristol, Bristol, BS8 2BN, UK and Population Health Sciences, Bristol Medical School, University of Bristol, Bristol, BS8 2BN, UK; d Division of Trauma and Orthopaedic Surgery, University of Cambridge, Box 180, Addenbrooke's Hospital, Hills Road, Cambridge CB2 2QQ, UK; e School of Biological Sciences, University of East Anglia, Norwich NR4 7TJ, UK

**Keywords:** microRNA, Cartilage, Osteoarthritis, Osteogenesis, Hedgehog signalling, Glypicans, SILAC

## Abstract

The Hedgehog (Hh) signalling pathway plays important roles during embryonic development and in adult tissue homeostasis, for example cartilage, where its deregulation can lead to osteoarthritis (OA). microRNAs (miRNAs) are important regulators of gene expression, and have been implicated in the regulation of signalling pathways, including Hh, thereby impacting upon development and disease. Our aim was to identify the function of miRNAs whose expression is altered in OA cartilage. Here we identified an increase in miR-324-5p expression in OA cartilage and hypothesised that, as in glioma, miR-324-5p would regulate Hh signalling. We determined that miR-324-5p regulates osteogenesis in human mesenchymal stem cells (MSCs) and in mouse C3H10T1/2 cells. Luciferase reporter assays demonstrated that miR-324-5p directly regulated established targets GLI1 and SMO in human but not in mouse, suggesting species-dependent mechanism of Hh pathway regulation. Stable Isotope Labelling with Amino acids in Cell culture (SILAC), mass spectrometry and whole genome transcriptome analysis identified Glypican 1 (Gpc1) as a novel miR-324-5p target in mouse, which was confirmed by real-time RT-PCR, immunoblotting and 3′UTR-luciferase reporters. Knockdown of Gpc1 reduced Hh pathway activity, and phenocopied the effect of miR-324-5p on osteogenesis, indicating that miR-324-5p regulates Hh signalling in mouse via direct targeting of Gpc1. Finally, we showed that human GPC1 is not a direct target of miR-324-5p. Importantly, as well as identifying novel regulation of Indian Hedgehog (Ihh) signalling, this study demonstrates how a miRNA can show conserved pathway regulation in two species but by distinct mechanisms and highlights important differences between human diseases and mouse models.

## Graphical abstract

**Figure:**
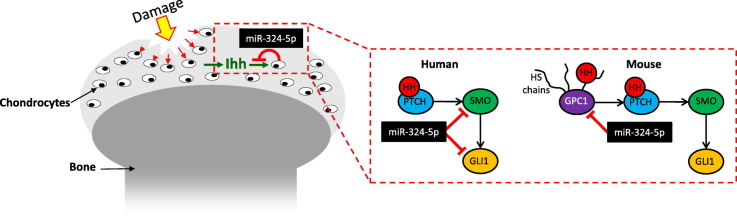

## Introduction

The Hedgehog (Hh) signalling pathway is important in embryonic development and adult tissue homeostasis and is aberrantly activated in many human diseases, including osteoarthritis (OA) [1]. The pathway can be activated by one of three Hh ligands, Sonic Hedgehog (Shh), Indian Hedgehog (Ihh) or Desert Hedgehog (Dhh), all via their binding to the membrane receptor Ptch. This binding results in loss of inhibition on the Hh signal transducer SMO, and accumulation of active Hh transcription factor Gli1. Gli1 induces the transcription of a number of Hh responsive genes including itself, *Ptch1* and *Hhip* [2]. During skeletogenesis Ihh functions to regulate chondrocyte differentiation and proliferation [3,4] by, for example, stimulating parathyroid hormone related peptide (PTHrP) expression to induce chondrocyte proliferation and to prevent hypertrophy during endochondral ossification [5]. Conversely, forced expression of Shh in chondrocytes during development leads to joint fusion [6]. In humans, mutations within IHH are known to cause Brachydactyly type A1 [7] and Acrocapitofemoral Dysplasia [8], two diseases involving skeletal abnormalities and short stature. Mice lacking Ihh also exhibit a short-limb dwarfism phenotype [9].

OA is the most common musculoskeletal disease and is epitomised by the loss of articular cartilage, thickening of subchondral bone and the formation of osteophytes [[10], [11], [12]]. The biological activity of the cartilage cell, the chondrocyte, during OA involves re-initiation of skeletal development pathways [13], including Hh signalling. Hh signalling is thought to contribute to the loss of cartilage by controlling expression of cartilage degrading enzymes such as matrix metalloproteinase (MMPs) [14], increasing chondrocyte hypertrophy [14], the formation of osteophytes, and thickening of subchondral bone. Mouse models have shown alteration in Hh activity alone can lead to early-onset-OA-like disease [1]. Additionally, blockade of Hh signalling in both surgical and serum-induced mice models of OA can be protective [1,15]. Similarly, deletion of Ihh in mice can protect against OA, consistent with Hh signalling contributing to OA pathogenesis [1,16]. At the cellular level, knockdown of IHH in human chondrocytes decreases both COL10A1 and MMP13 expression while the addition of exogenous Ihh increases expression [14]. However, activation of Hh signalling also leads to increased collagen II expression in rat meniscus [17] illustrating tight regulation of the Hh pathway is required for cartilage homeostasis.

MicroRNAs (miRNAs) are small (approximately 22 nt), single stranded, non-coding RNAs that modulate gene expression through base-specific interactions within the target gene 3′untranslated region (UTR) causing translational inhibition and mRNA degradation [18]. Numerous miRNAs influence the expression of matrix components and matrix remodeling in various diseases [19]. Through the generation of conditional cartilage-specific Dicer null mice, the overall importance for miRNAs in skeletogenesis has been demonstrated [20]. The mice, which lack virtually all cartilage miRNAs, display severe developmental defects due to a decrease in chondrocyte proliferation and a faster onset of hypertrophy [20], processes in which Ihh is known to play a role. Conditional removal of Dicer in cells expressing Shh in earlier limb development (with a Shh-Cre recombinase) also causes alterations in limb morphogenesis [21]. A number of miRNAs are implicated in chondrocyte biology; in particular miR-140 and miR-455 appear largely cartilage-specific while others regulate pathways critical to chondrocyte function [22]. miR-140 is highly expressed in cartilage and mice which lack the miR-140 locus exhibit smaller stature, skeletal development abnormalities and are more susceptible to OA, illustrating the role of miRNAs in maintaining chondrocyte function. Cartilage profiling studies have identified a number of additional miRNAs differentially regulated in OA [23,24].

Here we initially screened for differential microRNA expression in normal (neck of femur fracture; NOF) and OA cartilage, identifying miR-324-5p as upregulated in the diseased tissue which had previously been associated with regulation of Hh signalling. Next we used quantitative proteomic and transcriptomic microRNA target identification to investigate the mechanisms by which OA-associated miR-324-5p regulates the hedgehog signalling pathway in humans and mice.

## Results

### miR-324-5p is increased in end-stage osteoarthritic cartilage and targets components of the Hh pathway in human cells

**Fig. 1:**
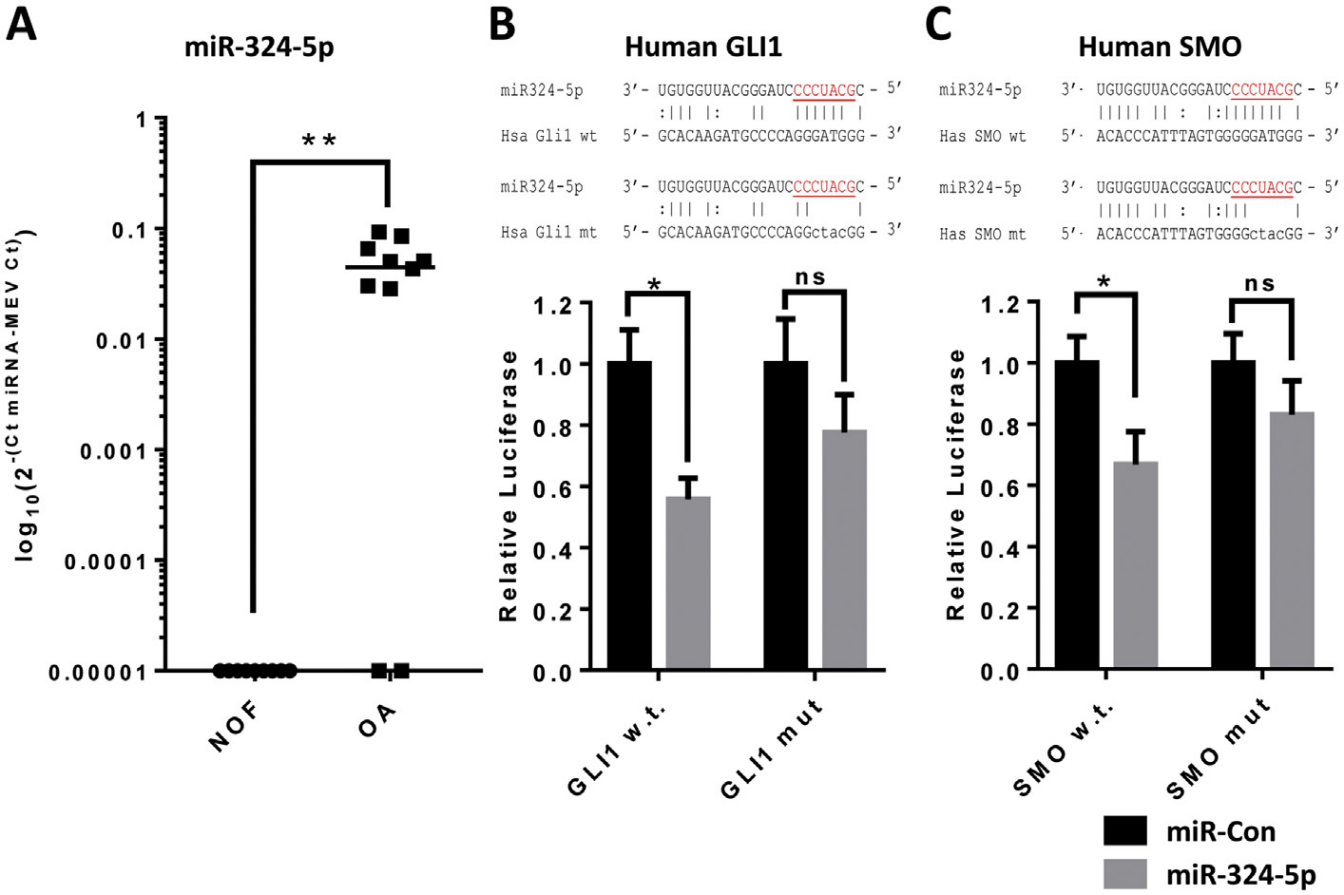
miR-324-5p expression is increased in OA and targets components of the Hh pathway in humans. (A) Human articular cartilage samples obtained from femoral heads of patients with OA (*n* = 10) were compared with those from patients undergoing hip replacement following fracture to the neck of femur (NOF; *n* = 10). RNA was purified, reverse transcribed and assayed by qRT-PCR for miR-324-5p expression. Statistical differences were calculated using an unpaired *t*-test with Welch's correction where **p < 0.01. (B and C) Schematic of miR-324-5p interaction with either wild-type (wt) or mutant (mt) human GLI1 and SMO 3′UTR luciferase constructs. Seed sequence is underlined and in red. SW1353 cells were transfected with either human GLI1 wild-type (w.t.), GLI1 mutant (mut), SMO w.t. or SMO mut 3′UTR luciferase constructs (in pMIR-Report) with either miCon2 or miR-324-5p, data plotted as relative luciferase light units. Data were combined from 5 independent experiments each *n* = 6. Statistical difference were calculated using a two-way analysis of variance followed by Bonferroni post hoc test for multiple comparisons where *p < 0.05, ns = non-significant (*p* > 0.05). Data are presented as standard error of the mean (SEM).

To identify miRNAs differentially expressed in OA, a TaqMan® low density array of 365 miRNAs was performed on cartilage RNA. Samples were obtained from total hip replacements for either OA or fracture to the neck of femur (NOF). Data were normalised to the mean expression value of the miRNAs profiled, using a previously published method [25]. A number of miRNAs were differentially expressed [26]. One miRNA of particular interest was miR-324-5p, whose expression was only detectable in the majority of OA, but not the control (NOF), cartilage (Fig. 1A).

Ferretti et al. [27] previously reported that miR-324-5p regulated Hh signalling in human medulloblastoma via direct targeting of the GLI1 and SMO 3′UTR. To confirm this in chondrocytes, we similarly cloned the human 3′UTRs of GLI1 and SMO, placing the expression of luciferase under their control. In agreement, co-transfection of miR-324-5p reduced the luciferase activity of both constructs (Fig. 1B and C). Furthermore, mutation of the putative miR-324-5p complementary seed sites within both the GLI1 and SMO 3′UTRs prevented miR-324-5p from exerting its repressive effects on the constructs (Fig. 1B and C), confirming miR-324-5p acts directly on the human SMO and GLI1 3′UTR, as proposed in medulloblastoma [27]. Interestingly, the expression of SMO and GLI are both also increased in OA cartilage (Supplementary Fig. 1), suggesting miR-324-5p is part of a larger regulatory network of Hh signalling.

### miR-324-5p regulates osteogenesis in human bone marrow derived mesenchymal stem cells

**Fig. 2:**
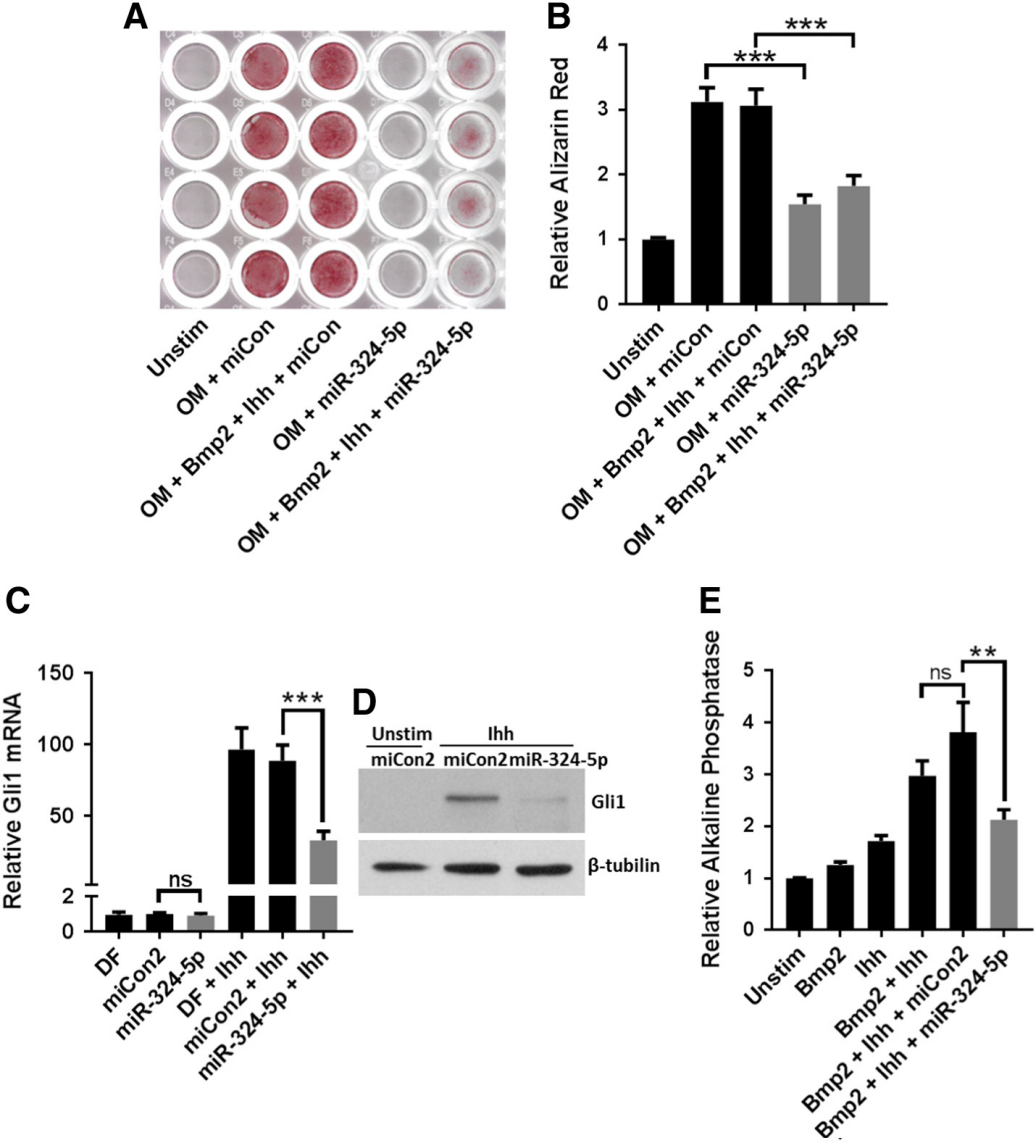
miR-324-5p regulates osteogenesis in human cells and both hedgehog signalling and osteogenesis in mouse C3H10T1/2 cells. (A and B) Human MSCs were transfected with either miCon or miR-324-5p (100 nM) for 2 days prior to OsteoMax-induced osteogenic differentiation in the presence or absence of Ihh (2 μg/ml) and BMP2 (100 ng/ml). Representative image from 1 donor following staining with Alizarin Red (*n* = 4/treatment) (A). Alizarin red was extracted using cetylpyridinium chloride and absorbance measured at 620 nm. Data were combined from 3 donors, each *n* = 4 and presented as standard error of the mean (SEM). C3H10T1/2 cells were transfected with miR-324-5p (100 nM) and stimulated with recombinant Ihh (2 μg/ml). Gli1 expression was measured using real-time PCR (C) and immunoblotting (D). (E) C3H10T1/2 cells were transfected with miCon2, miR-324-5p or exposed to transfection reagent alone and then stimulated with Ihh (2 μg/ml) and/or BMP2 (100 ng/ml) for a period of 5 days. *p*-nitrophenylphosphate (pNPP) was measured to determine the level of alkaline phosphatase as an indicator of osteoblastic differentiation. Data shown are combined data from 5 independent experiments each *n* = 3. Data are presented as standard error of the mean (SEM). Throughout statistical differences were calculated using a one-way analysis of variance followed by Bonferroni post hoc test where **p < 0.01, ***p < 0.001, ns = non-significant (*p* > 0.05).

The Hh signalling pathway is known to be involved in bone formation [28]. We therefore hypothesised miR-324-5p was also involved in this process. Overexpression of miR-324-5p in human bone marrow derived mesenchymal stem cells (MSCs) reduced the level of Alizarin Red staining following osteogenic induction, both in the presence and absence of additional BMP2 and Ihh, indicating miR-324-5p can regulate osteogenesis in human cells (Fig. 2A and B).

### miR-324-5p regulates Hedgehog signalling and osteogenesis in mouse C3H10T1/2

Since the miR-324-5p sequence is perfectly conserved between humans and mice, we also used the mouse pluripotent cell-line C3H10T1/2 to test the functional activity of miRNA, since under the correct stimuli these cells have the ability to undergo osteogenic differentiation [29]. Gli1, a Hh signalling transcription factor whose expression also increases following Hh stimulation as part of a positive feedback loop, was used as a readout of Hh signalling activity. Overexpression of miR-324-5p inhibited Ihh-induced Gli1 mRNA and protein expression (Fig. 2C and D), demonstrating that miR-324-5p can regulate Hh signalling in mouse C3H10T1/2 cells. BMP2 and Ihh can induce high levels of alkaline phosphatase in C3H10T1/2, indicative of osteogenesis [29] (Fig. 2E). Consistent with the effect on Hh signalling, overexpression of miR-324-5p reduced the level of Ihh and BMP2-induced alkaline phosphatase (Fig. 2E).

### miR-324-5p targets differ in human and mouse

**Fig. 3:**
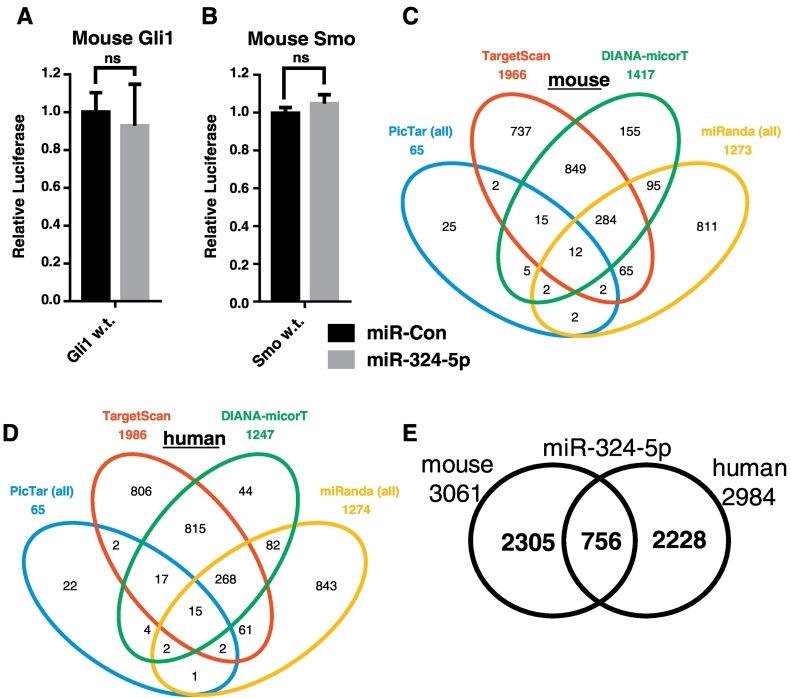
miR-324-5p targets differ in human and mouse. (A and B) pMIR report plasmid containing either mouse Gli1 or Smo 3′UTR downstream of luciferase was transfected into C3H10T1/2 cells with either miCon2 (miR-Con) or miR-324-5p. Data were normalised to miCon2 and plotted as relative luciferase light units. Data were combined from 3 independent experiments and presented as SEM. No differences were observed using a Student's *t*-test (*p* > 0.05). (C and D) miR-324-5p target identification by 4 commonly used target prediction software for mouse and human. (E) Crossover of miR-324-5p target prediction between mouse and human.

To test if miR-324-5p regulated IHH signalling via direct targeting of Gli1 and Smo in mouse we generated mouse specific 3′UTR luciferase constructs to recapitulate the effect miR-324-5p has in the human system. However, miR-324-5p did not repress activity from either reporter (Fig. 3A and B), despite the clear regulation of Hh signalling in the mouse C3H10T1/2 cell line (Fig. 2C and D). Examination of the mRNA sequences revealed neither murine Gli1 nor Smo 3′UTR contained any highly seed-complementary (‘8mer’, ‘7mer-m8’ or ‘7mer-1a’) binding sites for miR-324-5p. Together, these data would suggest that miR-324-5p regulates Hh signalling in human and mouse via differing mechanisms. To elucidate alternative potential targets of miR-324-5p in mouse that may regulate Hh signalling we combined data from 4 commonly used prediction algorithms; PicTar [30], TargetScan [31], DIANA-microT [32] and miRanda [33] giving a total of 3061 different predicted targets, however there were only 12 targets in common between databases (Fig. 3C), none of which had a known role in Hh signalling. A similar number of human targets were also predicted (Fig. 3D), with the majority of predicted targets being species specific (Fig. 3E). Given the large number and range of predicted targets and lack of consistency between prediction algorithms, we sought to identify murine miR-324-5p targets involved in Hh signalling experimentally.

### Identification of direct miR-324-5p targets

**Fig. 4:**
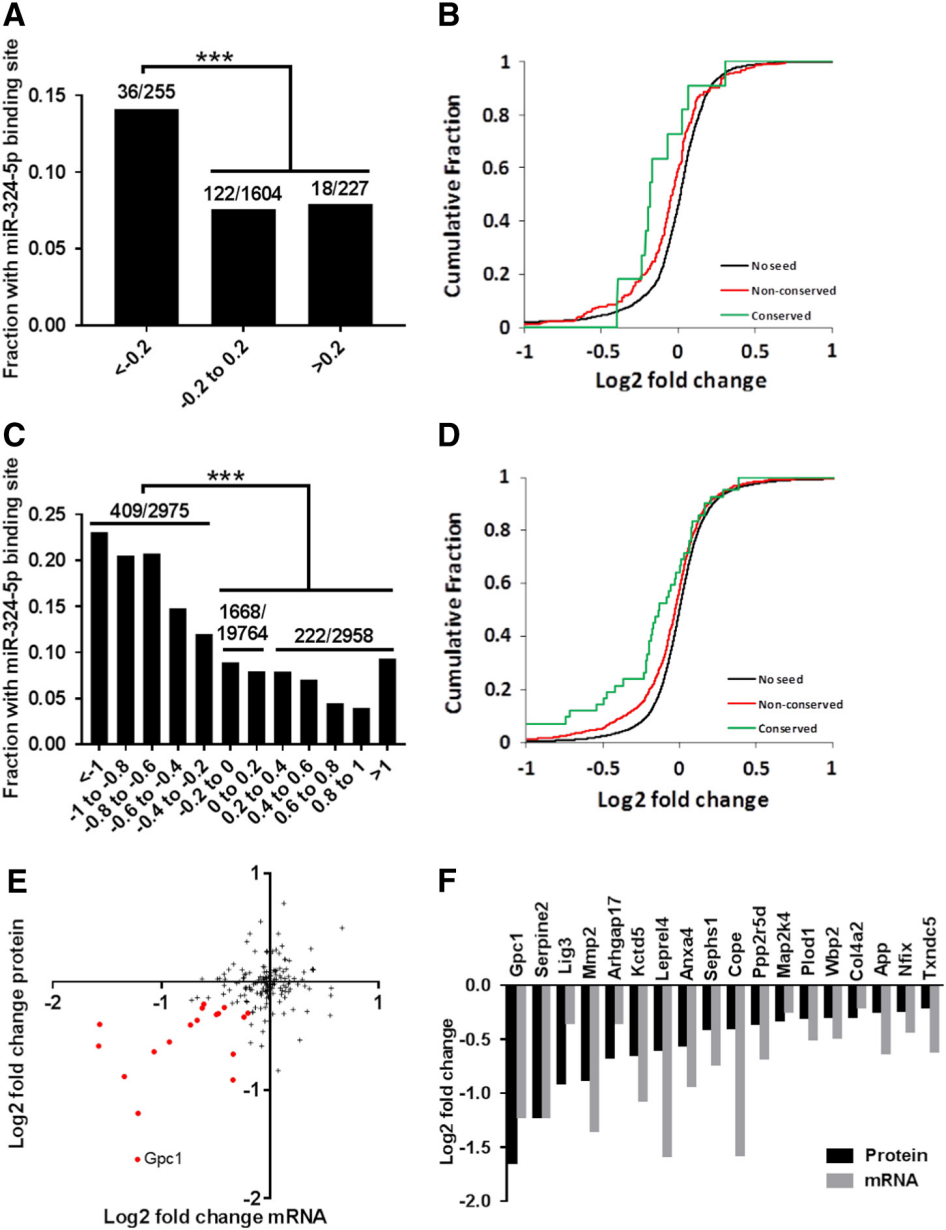
miR-324-5p target identification in Ihh treated mouse C3H10T1/2. (A) Enrichment of genes containing 3′UTR miR-324-5p binding sites within genes whose protein decreased (<log2 −0.2) following miR-324-5p transfection. (B) Cumulative fraction plot showing enrichment of conserved and non-conserved miR-324-5p binding site containing genes whose protein expression decreased following miR-324-5p over expression. (C) Enrichment of genes containing 3′UTR miR-324-5p binding sites within genes whose RNA decreased (<log2 −0.2) following miR-324-5p transfection. (D) Cumulative fraction plot showing enrichment of conserved and non-conserved miR-324-5p binding site containing genes whose transcript expression decreased following miR-324-5p over expression. (E) Proteomic and transcriptomic expression for 176 genes identified at a protein and mRNA level and also contain a miR-324-5p seed binding site, 18 of which decreased at an mRNA and protein level (highlighted in red). (F) Bar chart showing log2 fold change in protein and RNA expression for the 18 potential targets following miR-324-5p transfection. Enrichment significance for miR-324-5p binding site containing genes was calculated using Chi-squared test, where ***p < 0.001.

We used stable isotope labelling with amino acids in cell culture (SILAC) quantitative proteomics [34] to identify target proteins of miR-324-5p in Ihh-stimulated C3H10T1/2 cells. Following miR-324-5p overexpression, 255 proteins were decreased and 227 were increased, 36 and 18 of which contain a predicted miR-324-5p target site respectively. This finding indicates an enrichment of miR-324-5p target site containing genes in the proteins whose expression decreased following miR-324-5p overexpression (Fig. 4A). This enrichment was strongest for conserved targets, at log2 −0.2 cut-off (Fig. 4B), suggesting SILAC proteomics as a valid tool to identify direct miR-324-5p targets.

Overexpression of miRNAs followed by transcriptome profiling (RNA hybridisation microarray) has also previously been used to screen for miRNA targets [35]. Therefore, using similar parameters to our SILAC screen, we overexpressed miR-324-5p and stimulated C3H10T1/2 cells with Ihh. Following miR-324-5p overexpression, 2975 and 2958 transcripts were decreased and increased respectively, 409 and 222 of which contain a predicted miR-324-5p target respectively. As previously, this represents an enrichment of miR-324-5p target site containing genes in those whose transcript expression decreased following miR-324-5p mimic transfection (Fig. 4C). Once more, there was a greater enrichment of conserved targets over non-conserved targets (Fig. 4D). Pathway analysis of the 200 most decreased transcripts following miR-324-5p over-expression identifies an enrichment of genes associated with the extracellular matrix (ECM) and developmental proteins (Supplementary Table 1), suggesting an important role for miR-324-5p in such processes. Interestingly, enrichment in Wnt signalling pathway associated genes was also detected (Supplementary Table 1) illustrating the close relationship between Hh and Wnt signalling.

In total, 2086 genes were identified in both the transcriptome and proteomic screens, 176 of which contain a known miR-324-5p binding site in their 3′UTR (Fig. 4E). For 18 of these genes, expression decreased at both transcript and protein level by greater then log2 0.2 (Fig. 4F) and thus could be considered as potential miR-324-5p targets. None of these 18 potential targets are within the Hh KEGG pathway [36]. The most repressed potential target identified was Glypican 1 (Gpc1). Although Gpc1 is not found within the Hh KEGG pathway, family members of Gpc1 have been shown to play a role in Hh signalling [[37], [38], [39]]. We therefore hypothesised that Gpc1 could be a target of miR-324-5p in mouse, regulating Hh signalling.

### Gpc1 is a direct miR-324-5p target and regulator of Hh signalling in mouse

**Fig. 5:**
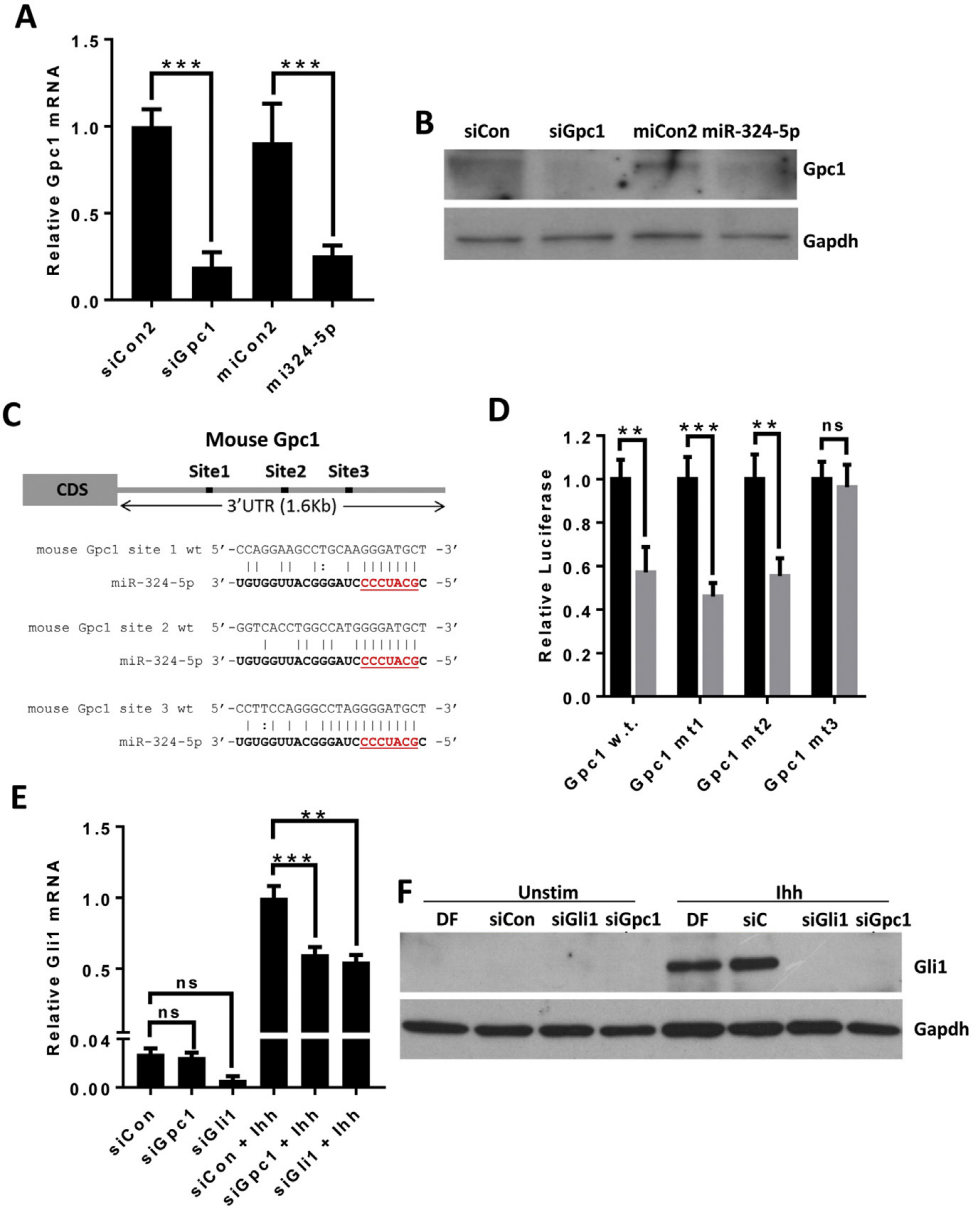
miR-324-5p targets Gpc1 to regulate Hh signalling in mouse C3H10T1/2. (A) Real time RT-PCR analysis of Gpc1 mRNA following siGpc1 or miR-324-5p transfection. Data are presented as SEM. (B) Western blot analysis of Gpc1 protein following siGpc1 and miR-324-5p transfection. (C) Schematic showing potential interaction of miR-324-5p with 3 different binding sites in mouse Gpc1 3′UTR. (D) pMIR reporter plasmid containing either wild type, mutant site 1, mutant site 2 or mutant site 3 mouse Gpc1 3′UTR down stream of luciferase was transfected into C3H10T1/2 cells with either miCon2 or miR-324-5p. Data were normalised to miCon2 and plotted as relative luciferase light units. Data were combined from 5 independent experiments, each *n* ≥ 6. (E) siGli1 and siGpc1 effect on basal and Ihh stimulated Gli1 mRNA expression. C3H10T1/2 cells were transfected with non-targeting siCon or siRNA against Gpc1 or Gli1 for 24 h. Cells were then serum starved for 24 h and either left unstimulated or stimulated with Ihh (2 μg/ml) for 24 h. Data were combined from 4 independent experiments, each *n* = 4. (F) siGli1 and siGpc1 effect on basal and Ihh stimulated Gli1 protein expression, cells treated as in D. Representative blot of 3 independent experiments. Throughout statistical differences were calculated using a one-way analysis of variance followed by Bonferroni post hoc test where **p < 0.01, ***p < 0.001, ns = non-significant (*p* > 0.05).

Targeting of Gpc1 mRNA and protein by miR-324-5p was confirmed in mouse C3H10T1/2 cells (Fig. 5A and B). Next, we validated Gpc1 as being a direct target of miR-324-5p using a mouse Gpc1 3′UTR-luciferase construct (Fig. 5C). Further analysis of the mouse Gpc1 3′UTR identified 3 potential miR-324-5p seed matches (all ‘7mer-m8’). Site-directed mutagenesis of each seed matched sequence indicated only the most distal 3′ site (here named as site 3) is functional (Fig. 5D).

To test if Gpc1 is important for Hh signalling, C3H10T1/2 cells were depleted of Gpc1 using siRNA transfection and stimulated with Ihh, again Gli1 transcript and protein was used as a readout for Hh pathway activation. As with miR-324-5p transfection (Fig. 2C and D), siGpc1 transfected cells showed reduced levels of Gli1 following stimulation (Fig. 5E and 5F), indicating Gpc1 is required for Ihh signalling in murine cells.

### miR-324-5p does not target human GPC1

**Fig. 6:**
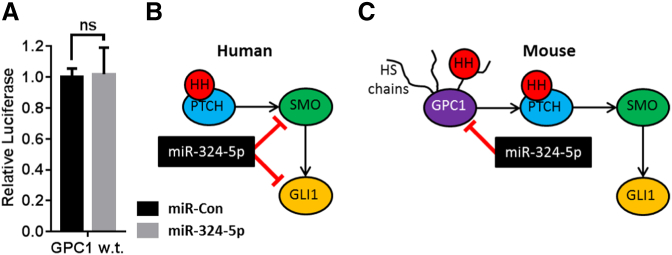
miR-324-5p regulates Hh signalling by different mechanisms in human and mouse. (A) pMIR reporter plasmid containing human GPC1 3′UTR downstream of luciferase was transfected into SW1353 cells with either miCon2 or miR-324-5p. Data shown were normalised to miCon2 and plotted as relative luciferase light units. No difference was observed between groups using Student's *t*-test (*p* = 0.92). Data are presented as SEM. (B and C) Schematic showing proposed miR-324-5p regulation of Hh signalling in human and mouse.

Analysis of the human GPC1 3′UTR identified one centrally located putative miR-324-5p seed binding site, though this was a relatively poor ‘7mer’. A human GPC1 3′UTR luciferase construct was then created, expression was surprisingly unaffected by miR-324-5p (Fig. 6A), suggesting that human GPC1 is not a direct target of miR-324-5p.

## Discussion

Here we show miR-324-5p regulation of Hh signalling is conserved in humans and mice, yet the regulatory mechanism is not. In order to regulate Hh signalling in humans, miR-324-5p targets SMO and GLI1, but not GPC1, whereas in mice, miR-324-5p targets Gpc1, but not Smo or Gli1 (Fig. 6B and C).

Initially to identify miRNAs differentially expressed in OA, we screened end-stage OA articular cartilage and used healthy articular cartilage from Neck of femur fracture (NOF) patients as controls. These sample types have been previously used to identify differences between OA and healthy cartilage [40]; including miRNA expression differences [26]. This study is the first to show miR-324-5p is differentially expressed in OA cartilage; in fact miR-324-5p was only detected in OA cartilage. Although lowly expressed, miR-324-5p has previously been identified in cartilage [41,42]. Crowe et al. found 0.003% of all miRNA RNA-seq reads mapped to miR324-5p, which represented the 172nd (out of 990 identified) most abundant miRNA in OA cartilage [41]. Hasseb et al., also identified miR-324-5p in isolated primary chondrocytes, however was only the 319th (out of 437 identified) most abundant miRNA and represented only 0.0003% of all miRNA reads [42].

Given the role of Hh signalling in OA [1], the data herein expands knowledge on regulation of Hh signalling during OA. Hh signalling activity is increased during human OA and in the destabilization of the medial meniscus (DMM) mouse OA model [1], indicative of developmental pathway reinitiation. Hedgehog inhibition protects cartilage against OA (SMO inhibition reduces hypertrophy and osteophyte formation) in mice [15]. Interestingly, the expression of miR-324-5p (here a potent Hh inhibitor), SMO and GLI1 (human targets) and GPC1 (mouse target) are all increased in our human OA dataset, suggesting miR-324-5p plays an important part of a complex hedgehog regulatory network. We hypothesise increased miR-324-5p is part of a failed attempt by the chondrocytes to reduce Hh signalling, however, future studies to modulate the levels of miR-324-5p in models of OA, are needed to determine if it has a positive or negative impact on OA severity.

MiRNAs often have many targets, with both miRNA and target requiring temporo-spatial co-expression for effective regulation. Therefore to identify miR-324-5p targets involved in Hh regulation, C3H10T1/2 were stimulated with Ihh, ensuring potential target mRNA important for Hh signalling regulation were expressed. Since the majority, of miRNA targets are decreased at an mRNA and protein level [43], we combined both transcriptome microarray and quantitative proteomics (SILAC) data to identify Gpc1 as a potential miR-324-5p target. The mouse Gpc1 3′UTR contained three potential miR-324-5p binding sites, however, only the most distal site was functional, supporting the observation that sites closer to the ends of the 3′UTRs are more effective targets [44,45].

Members of the glypican family have been reported to regulate Hh signalling. For example, Gpc3 in mice and Dally-like protein in *Drosophila* are described as being involved in Hh signalling [37,46]. Data has linked GPC1 with Shh in Biliary atresia [47] and importantly identified GPC1 as a Shh receptor in commissural neurons [48]. Glypicans are a class of Glycosylphosphatidylinositol (GPI) anchored membrane heparin sulphate proteoglycan (HSPG) consisting of a core protein with chains of heparan sulphate (HS) glycos-amino-glycan (GAG) chains [19,49]. HSPGs, such as glypicans, can interact with morphogens to produce and regulate morphogen gradients, for review see [50]. Thus it is suggested glypicans function in Hh signalling by either sequestering or presenting Hh ligands via their HS chains to Hh receptor Ptch. An interesting observation in our SILAC proteomics experiment was that transfection of miR-324-5p caused a reduction in the amount of added Ihh protein detected within the cell lysate (Supplementary Fig. 2). This indicates that over-expression of miR-324-5p and subsequent reduction in Gpc1 reduce the capability of the cell to uptake, present or retain Ihh ligand as suggested previously for Shh [51]. Glypican family members are known to regulate Hh signalling within the growth plate [52], potentially suggesting a role for miR-324-5p during rodent limb development as well as during OA.

HS is a well-established regulator of cartilage development and maintenance [49,53]. Consistent with this, before directly confirming a role for Gpc1 in Hh signalling, we cultured cells in sodium chlorate, which inhibits HS formation, and showed this reduced Ihh-induced Gli1 expression, confirming an overall role for HS in Ihh signalling (Supplementary Fig. 3). Interestingly, transient overexpression of Gpc1 also inhibited Hh signalling (Supplementary Fig. 4) indicating a Goldilocks zone. Indeed, glypicans have previously also been shown to interact with multiple morphogens to both promote and inhibit cell signalling, for example, Gpc3 can directly interact with Shh to cause endocytosis and lysosomal degradation leading to Hh signalling inhibition [37]. Gpc3 can also interact with Wnts to promote the Wnt signalling pathway [37,54]. When considered together with our pathway analysis, data may suggest miR-324-5p regulates Wnt signalling through a mechanism also involving Gpc1.

Genotype-phenotype correlations show Glypican 1 can be a cause of skeletal deformities in rodents and is highly expressed in developing and mature osteoblasts [55]. In humans the Glypican 1 gene has previously been identified as a possible candidate for causing Brachydactyly Type E, an inherited condition causing skeletal deformities [56]. miR-324-5p and Gpc1 are also highly expressed in the nervous system [56]. In humans, patients with a loss of the genomic locus containing GPC1 (chromosome 2q) display skeletal deformities and also mental retardation [56]. In mice Gpc1 deletion alters brain size and leads to increased risk of cancer, however animals show no skeletal phenotype, as is frequently reported for deleted matrix-associated genes; e.g. Matrilin-3, [57]. *Gpc1* is unlikely to be the sole mechanism by which miR-324-5p exerts any effects on the nervous system, and in fact we found that miR-324-5p can also inhibit *App* expression (Fig. 4F); a gene whose protein contributes to brain pathogenesis, such as in Alzheimer's disease.

From an evolutionary perspective the miR-324 stem loop is predicted to have arisen in the genome around 100 million years ago with the appearance of placental animals [58]. Given this age, it is interesting that miR-324-5p regulates Hh signalling in humans and mouse by potentially differing mechanisms, targeting human GLI1 and SMO and mouse Gpc1 to inhibit Hh signalling. We confirmed this differential regulation using 3′UTR luciferase assays which remain the standard technique for validating direct targets of miRNAs. We also found no evidence that miR-324-5p could regulate human GPC1 transcript levels. Our data are consistent with the notation that miRNA:target interactions are not fixed during evolution [59], although the overall impact of miR-324-5p is to regulate Hh in both species. As glypicans have been shown to regulate a number of other morphogens, it is possible miR-324-5p inhibition of Gpc1 in mice may serve as a function to regulate morphogen signalling on a more general level, rather than regulate the core Hh signalling genes as miR-324-5p does in human. Future studies should address the role miR-324-5p plays in cartilage development and maintenance in the context of hedgehog signalling for both mice and humans.

Our data illustrates miR-324-5p targets Gpc1 to regulate Ihh signalling and likely other morphogens in mice, whereas in humans miR-324-5p is a more direct regulator of Hh signalling. This study highlights the importance of using human cells to study human development and disease, particularly in the context of miRNAs and hedgehog signalling. In conclusion miR-324-5p expression is increased in OA cartilage compared to healthy cartilage and regulates the Hh signalling pathway in human and mouse, but does so via differing mechanisms.

## Materials and methods

### Differential expression of miRNAs in OA vs. normal cartilage

A differential expression screen of miRNAs in OA cartilage obtained from patients undergoing joint replacement and healthy cartilage obtained from patients following fracture of the neck of femur (NOF), was performed and analysed as previously described [26].

### Cell culture

SW1353 were cultured in Dulbecco's Modified Eagle's medium (Sigma), supplemented with 10% foetal bovine serum (FBS, Sigma-Aldrich), 2 mM l-glutamine, 1% non-essential amino acid solution, 100 IU/ml penicillin and 100 μg/ml streptomycin. Human MSC were cultured in mesenchymal stem cell growth medium (Lonza Biosciences) supplemented with 5 ng/ml fibroblast growth factor-2 (R&D Systems, Abingdon, U.K.). For all experiments involving C3H10T1/2 mouse pluripotent mesenchymal cells, with the exception of SILAC experiments, cells were cultured in Minimum Essential Medium, supplemented as described for SW1353 cells. All cells were cultured in vented T75 cm^2^ flasks at 37 °C and 5% (v/v) CO_2_.

### Plasmid construction

Potential target 3′UTRs were amplified from genomic DNA using PCR primers (Supplementary Table 2) for In-Fusion® cloning (Clontech) or double-restriction enzyme digestion (*Spe*I/*Hind*III) to enable cloning into the pMIR-Reporter vector (Ambion). Mutation of the miRNA seed binding sites was performed using QuickChange II (Agilent). Mouse Gpc1 overexpression plasmid was generated by infusion cloning of Gpc1 cDNA clone (Source Bioscience) into N-terminal HaloTag® expression construct (Promega).

### Plasmid, siRNA and miRNA transfections

SW1353 chondrosarcoma cells (for human 3′UTRs) or mouse C3H10T1/2 cells (for mouse 3′UTRs, siRNA and miRNA) were seeded to reach ~50% confluence after 24 h, representing 1.8 × 10^4^ cells/cm^2^ for SW1353, 1.2 × 10^4^ cells/cm^2^ for MSC and 9 × 10^3^ cells/cm^2^ for C3H10T1/2. miRNA and siRNA (50–100 nM) were transfected using Dharmafect transfection reagent 1 (DF1, Dharmacon). Expression and reporter plasmids (500 ng/ml) were transfected using FugeneHD. For miRNA and reporter plasmid co-transfection incubated lipid complexes were added to cells simultaneously, essentially as previously described [60]. Transfection reagents alone were used as controls. After the 24 h of transfection, cells were lysed and luciferase levels determined (Promega) using a Microlumat Plus Luminometer, or were cultured further for stimulation and differentiation experiments.

### Human MSC osteogenesis and Alizarin Red staining

Human MSCs were cultured and transfected with either miCon or miR-324-5p in 96 well plates as described above. After 48 h media was replaced with OsteoMax medium (Merck Millipore) in the presence or absence of Recombinant Indian Hedgehog (Ihh) (2 μg/ml; R&D systems) and BMP2 (100 ng/ml; R&D systems). Fresh OsteoMax medium was added after 4 days. After 7 days cells were fixed in 10% formalin and mineralisation assessed by incubation with Alizarin Red solution (Sigma) (40 mM, pH 4.2) for 30 min. For quantification the staining was extracted with 10% (w/v) cetylpyridinium (Sigma) solubilised in 10 mM sodium phosphate buffer (pH 7) and the absorbance measured at 620 nM.

### Cell stimulation

C3H10T1/2 cells (with or without transfection) of 80–90% confluence were serum-starved for 16 h and stimulated with either Ihh or BMP-2 for the time and concentration indicated in each figure.

### RNA isolation, reverse transcription and real-time PCR

Total RNA was extracted using Cells-to-cDNA II Kit (Ambion, Huntington, UK). Complementary DNA (cDNA) was synthesised from total RNA using MMLV reverse transcriptase and random hexamers according to the manufacturer's instructions (Life Technologies). mRNA levels were determined using TaqMan® real time PCR. Assay primers and probes are listed or numbered as in the universal probe library (Roche) in Supplementary Table 2. For miRNAs, cDNA was synthesised from total RNA using TaqMan® MicroRNA Reverse Transcription (Life Technologies) according to the manufacturer's instructions. Real-time PCR was carried out using Life Technologies miRNAs assays according to the manufacturer's instructions.

### Immunoblotting

Cells were lysed using ice cold lysis buffer containing: 50 mM Tris-HCl, 1.2 M glycerol, 1 mM EGTA, 1 mM EDTA, 1 mM Na_3_VO_4_, 10 mM 2-glycerophosphate, 50 mM NaF, 5 mM sodium pyrophosphate, 1% (v/v) Triton X-100, 1 μM microcystin-LR and 0.1% (v/v) 2-mercaptoethanol. Particulates were removed by centrifugation at 13,000*g* for 5 min, supernatants were frozen at −80 °C until needed. To determine protein concentration, Bradford reagent (Biorad) was added to the lysate, absorbance measured at 595 nm with a plate reader (TECAN) and protein concentrations equalised. Proteins (5 μg per sample) were separated by SDS-PAGE electrophoresis, transferred to nitrocellulose membrane and probed with antibodies against Gli1 (V812, Cell Signalling, 1/1000), Gpc1 (16700-1-AP, Protein Tech Group, 1/1000), GAPDH (Chemico International, 1/1000) or β-tubulin (1/1000). Expression was monitored by chemiluminescence (GE Healthcare) using HRP conjugated secondary antibodies (1/2000).

### Alkaline phosphatase assay

C3H10T1/2 cells were cultured and transfected as described above in 24 well plates. After 48 h the cells were stimulated as indicated (Fig. 2C) in serum containing medium for 5 days. Culture medium was refreshed at day 3, after which cells were assessed for alkaline phosphatase activity. Briefly, cells were washed with PBS, fixed with 4% (w/v) formaldehyde, washed with HEPES and alkaline phosphatase substrate (*p*-nitrophenylphosphate, Sigma) added. Active alkaline phosphatase (produced *p*-nitrophenol) was measured at 405 nm, using a TECAN plate reader and Xflor 4 software.

### SILAC and mass spectrometry

C3H10T1/2 cells were cultured for more than five population doublings (7 days of culture), in DMEM containing either isotopically labelled ^13^C l-Lysine-2HCl and ^13^C ^15^N l-Arginine-HCl (heavy), or normal Lysine and Arginine (light). Both heavy and light media contained dialysed FBS (all SILAC media, amino acids and FBS were supplied by Thermo Scientific). After 7 days of culture, the isotopic incorporation of the heavy amino acids was assessed. More than 99% of the peptides assessed by mass spectrometry contained heavy Arginine and Lysine (data not shown). Both heavy and light cells were seeded in 6 cm dishes to reach around 50% confluence after 24 h. Cells were then transfected using DF1 with 100 nM of either miCon2 (non-targeting miRNA mimic) or miR-324-5p mimic in the light and heavy cells, respectively. After 24 h of transfection, cells were serum-starved for a further 16 h and then stimulated with 2 μg/μl recombinant Ihh for 48 h. Cells were then lysed using 150 μl of lysis buffer, as described for immunoblotting. The Heavy and Light lysates were then mixed at a ratio of 1:1. Protein was boiled and separated using SDS-PAGE with nanopure H_2_O used in all gels and buffers. Gel preparation, protein digestion, mass spectrometry and initial processing of data was performed by NEPAF, Newcastle University. Gels were cut into 12 segments, each digested with trypsin and analysed by mass spectrometry. Data were normalised and analysed using MaxQuant software as described previously [61].

### Total RNA isolation and microarray

C3H10T1/2 cells were cultured as described and seeded into 6 well plates at a density of 8.2 × 10^4^ cells per well for 24 h. Cells were then transfected using DF1 and 100 nM of either miCon2 or miR-324-5p for 24 h. Cells were then serum starved for 16 h before being stimulated for a further 48 h as described. Cells were then lysed and RNA extracted using the Qiagen RNeasy mini kit according to the manufacturer's protocol. Microarray analysis used the Illumina mouse ref8 v3 bead array and was performed by The Genome Centre, Queen Mary, University of London. Raw expression data were analysed using Agilent GeneSpring GX 11 (Agilent Technologies, Santa Clara, CA, U.S.A.). Raw data were normalised with a quantile algorithm and the baseline was transformed to the median of all samples prior to further analysis.

### Seed enrichment analysis

Enrichment analysis was performed by calculating the fraction of genes containing miR-324-5p binding site within the 3′UTR, for decreased (FC < log2 −0.2), increased (FC > log2 0.2) and unaltered transcripts and proteins. The list of genes whose 3′UTR contained a miR-324-5p binding site was obtained from TargetScan.

Cumulative fraction plots were generated by plotting FC gene expression on the x axis against the fraction of all genes whose 3′UTR contain (or does not contain for control) a miR-324-5p binding site on the y axis for all genes whose expression decreased more than that value on the x axis. The list of genes containing non-conserved and conserved miR-324-5p binding sites was obtained from TargetScan [31].

### Statistical analysis

Statistical differences were calculated using a one- or two-way analysis of variance followed by Bonferroni post hoc test for multiple comparisons or Student's *t*-test performed for single comparisons. Enrichment was calculated using Chi-squared test. *p < 0.05, **p < 0.01, ***p < 0.001, ns = *p* > 0.05. Statistical test is indicated within figure legends.

## Appendix A Supplementary data

Supplementary material Image 1
