# Supplementary material for: miR-324-5p is up regulated in end-stage osteoarthritis and regulates Indian Hedgehog signalling by differing mechanisms in human and mouse
Source: Matrix Biol. 2019 Apr;77:87–100. doi: 10.1016/j.matbio.2018.08.009 (PMC6456721; doi:10.1016/j.matbio.2018.08.009)
Supplement: Supplementary file 1 — Supplementary material [file mmc1.pdf]

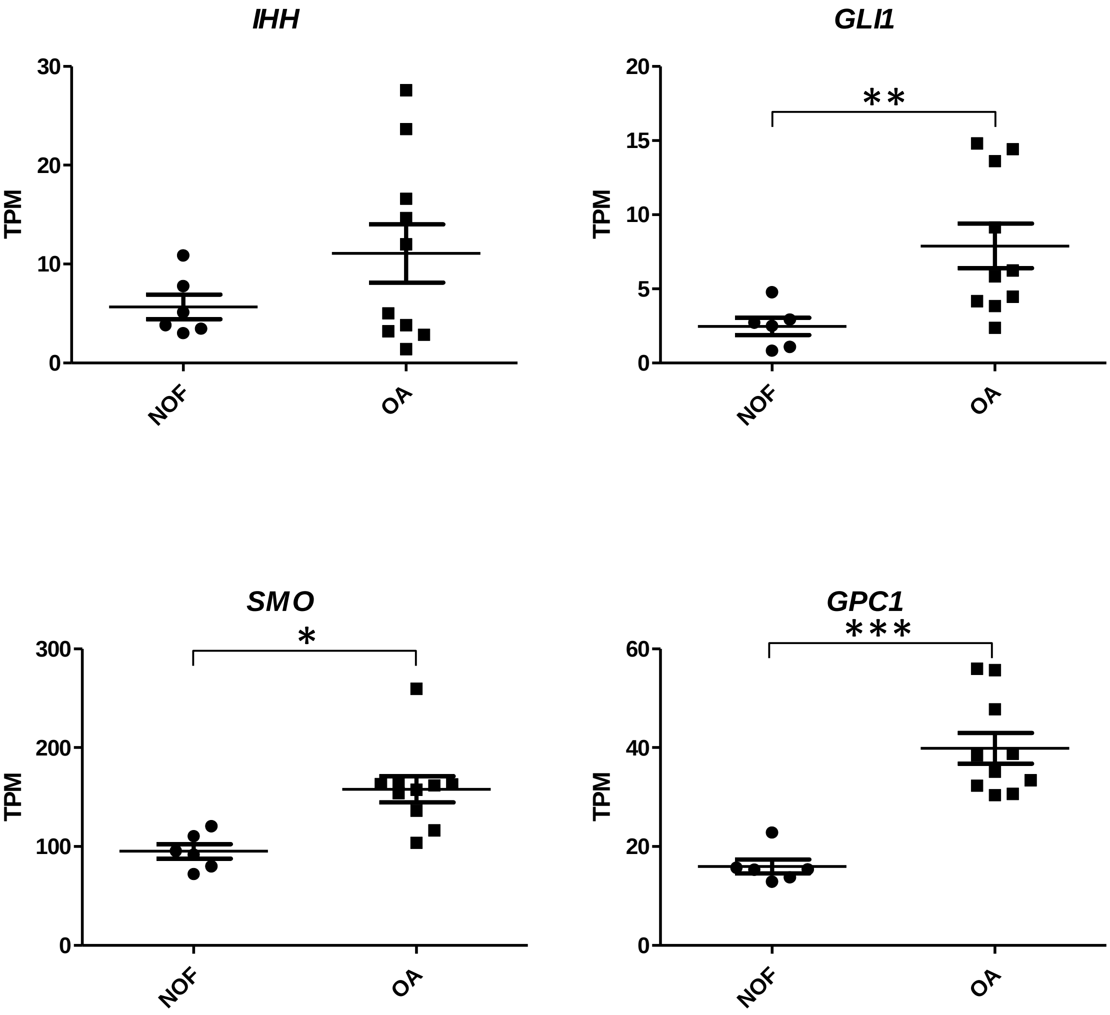

Supplementary figure 1. RNA-seq was performed on total RNA isolated directly from female femoral head articular cartilage samples (NOF, n=6; OA, n=10) as described Bui et al. 2012. Fastq files are available to download from the National Center for Biotechnology Information Expression Omnibus GSE111358. For the analysis, transcript counts were estimated using Salmon in quasi-mapping mode against human reference genome hg38 with default settings. DESeq2 was used for differential expression testing with whole dataset. FDR q values are given where * q<0.05; ** q <0.01 and *** q<0.001. TPM = transcripts per million.

Bui C, Barter MJ, Scott JL, Xu Y, Galler M, Reynard LN, et al. cAMP response element-binding (CREB) recruitment following a specific CpG demethylation leads to the elevated expression of the matrix metalloproteinase 13 in human articular chondrocytes and osteoarthritis. FASEB J 2012; 26: 3000-3011

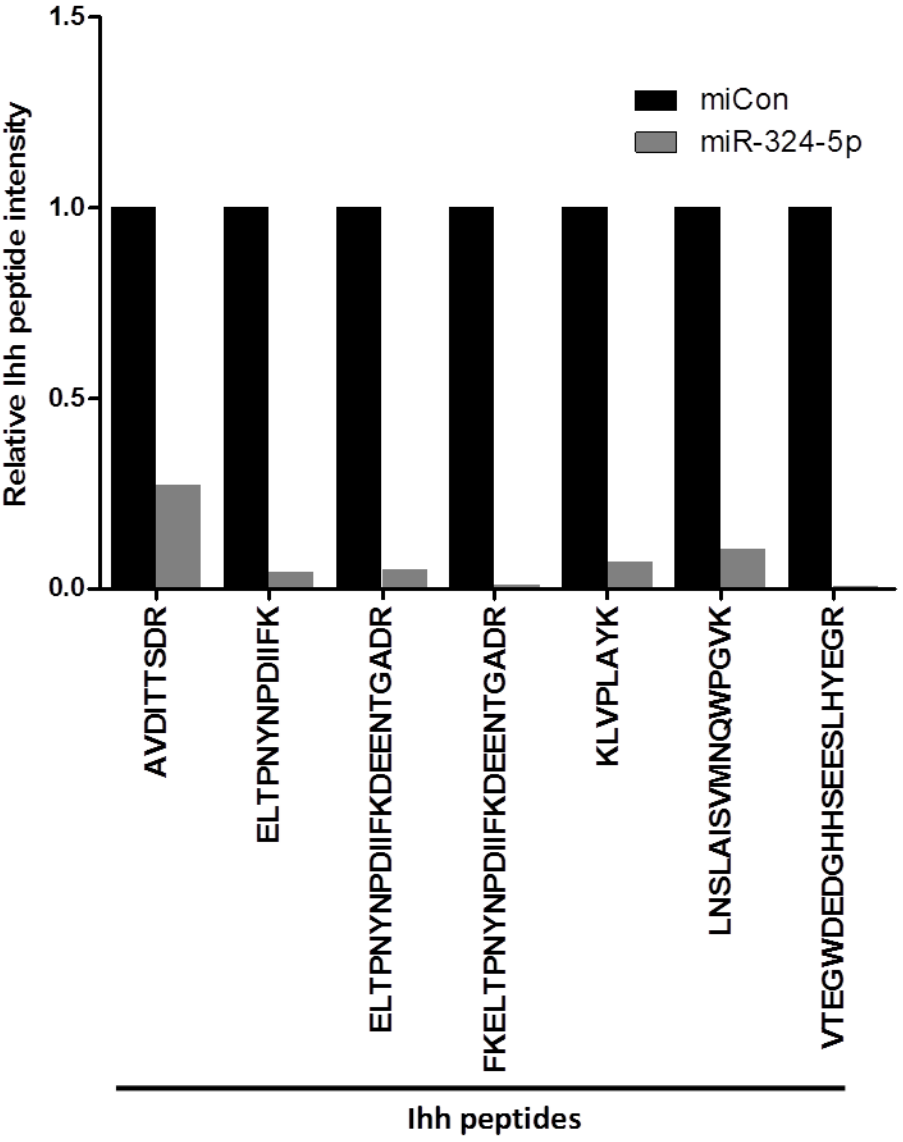

Supplementary figure 2. miR-324-5p decreases cellular Ihh. SILAC mass spectrometry detected fewer peptides associated with Ihh protein, following miR-324-5p transfection for 24h, followed by serum starvation for 24h, followed by stimulation with Ihh (2µg/ml) for 24h.

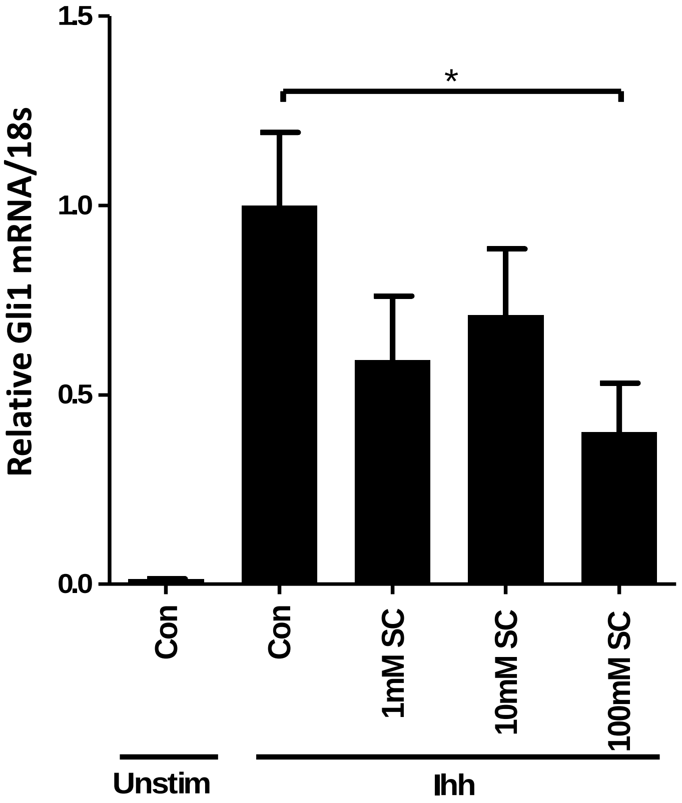

Supplementary figure 3. Sodium chlorate decreases Ihh signalling. C3H10T1/2 cells were treated with Sodium chlorate (SC) to prevent HS synthesis for 48h at the concentration shown, then serum starved for 24h and stimulated with Ihh (2µg /ml) for 24h. Data combined from 2 independent experiments, each n=3. Statistical differences were calculated using ANOVA followed by a Bonferroni post test, $p<0.05$=*.

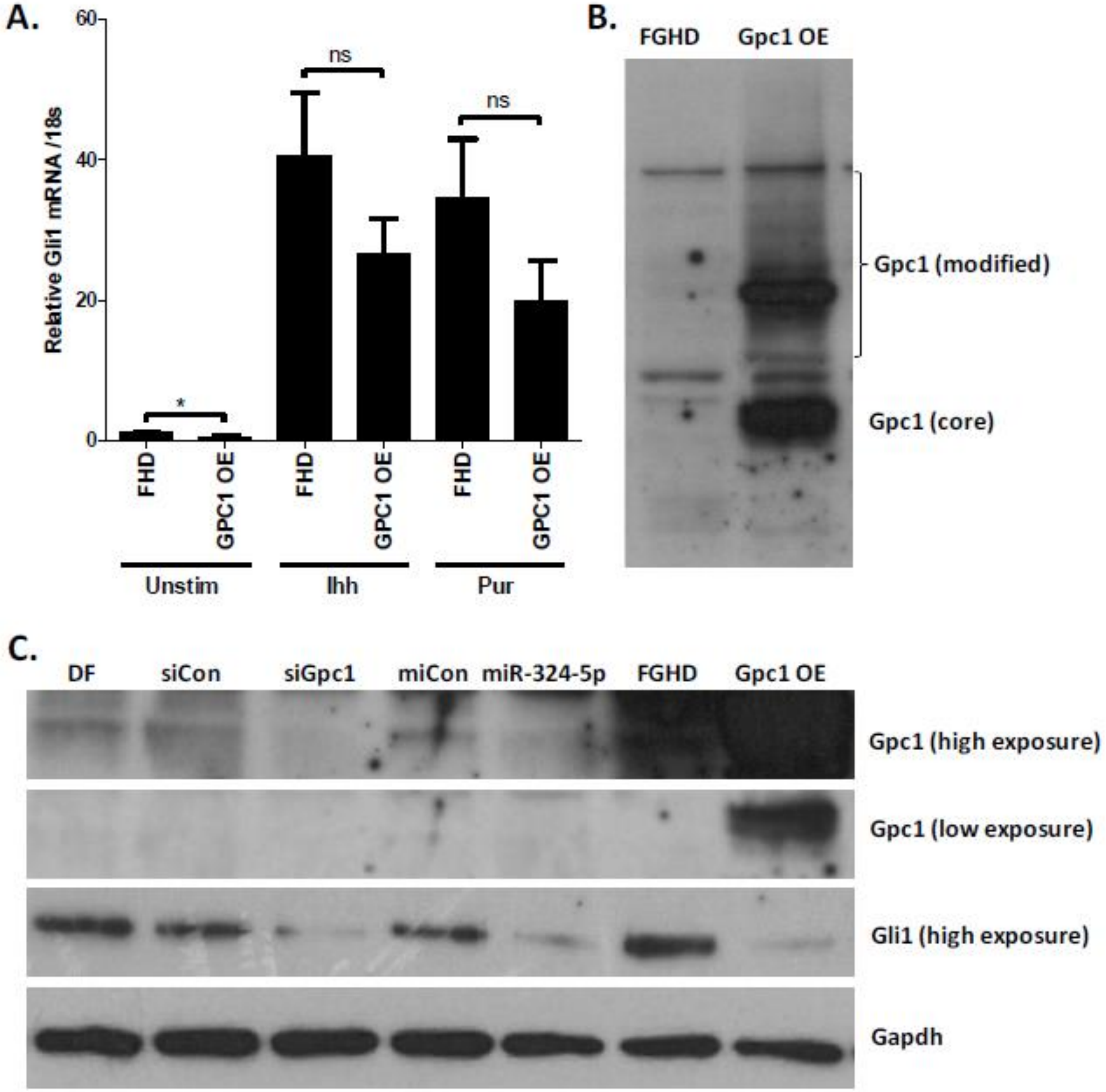

Supplementary figure 4. Overexpression of GPC1 reduces Ihh signalling. A) Gpc1 overexpression plasmid was transfected into C3H10T1/2 using Fugene HD (GPC1 OE) or mock transfected (FHD) for 24h, followed by serum starvation for 24h, followed by stimulation with Ihh (2µg/ml) or Pur (2µM) for 24h. Data combined from two independent experiments each n=4. Statistical difference were calculated using Student's t-test, where *$p<0.05$. (B) Example Western blot for Gpc1 following Gpc1 overexpression (OE) compared with Fugene HD transfection reagent alone (FGHD). (C) C3H10T1/2 were transfected with either siCon, siGpc1, miCon, miR-324-5p, Gpc1 overexpression construct or were mock transfected for 24h, followed by serum starvation for 48h. Western blotting was performed for Gpc1 and Gli1 protein.

Category	Term	RT	Count	%	P-Value	Benjamini
GOTERM_CC_DIRECT	proteinaceous extracellular matrix	RT	17	11.5	9.60E-10	1.90E-07
UP_KEYWORDS	Glycoprotein	RT	56	37.8	1.60E-09	2.90E-07
UP_KEYWORDS	Disulfide bond	RT	47	31.8	3.50E-08	3.10E-06
GOTERM_CC_DIRECT	extracellular region	RT	34	23	1.70E-07	1.60E-05
UP_KEYWORDS	Signal	RT	57	38.5	3.10E-07	1.80E-05
UP_KEYWORDS	Secreted	RT	30	20.3	1.10E-06	4.90E-05
UP_KEYWORDS	Extracellular matrix	RT	11	7.4	3.20E-06	1.20E-04
UP_SEQ_FEATURE	signal peptide	RT	49	33.1	7.40E-07	2.40E-04
UP_SEQ_FEATURE	glycosylation site:N-linked (GlcNAc...)	RT	54	36.5	4.00E-07	2.60E-04
COG_ONTOLOGY	Defense mechanisms	RT	4	2.7	7.00E-05	3.50E-04
GOTERM_CC_DIRECT	extracellular space	RT	28	18.9	6.70E-06	4.30E-04
UP_KEYWORDS	Wnt signaling pathway	RT	9	6.1	2.00E-05	6.00E-04
UP_SEQ_FEATURE	disulfide bond	RT	41	27.7	3.80E-06	8.20E-04
GOTERM_CC_DIRECT	extracellular matrix	RT	11	7.4	5.00E-05	1.90E-03
GOTERM_CC_DIRECT	cell surface	RT	16	10.8	4.30E-05	2.10E-03
UP_KEYWORDS	EGF-like domain	RT	9	6.1	1.10E-04	2.80E-03
UP_KEYWORDS	Calcium	RT	16	10.8	3.20E-04	7.10E-03
UP_KEYWORDS	Alternative splicing	RT	49	33.1	6.90E-04	1.40E-02
GOTERM_CC_DIRECT	membrane	RT	70	47.3	5.80E-04	1.90E-02
INTERPRO	EGF-like, conserved site	RT	9	6.1	7.50E-05	2.80E-02
UP_KEYWORDS	Developmental protein	RT	16	10.8	1.70E-03	3.00E-02
INTERPRO	Epidermal growth factor-like domain	RT	9	6.1	2.70E-04	3.40E-02
GOTERM_MF_DIRECT	heparin binding	RT	8	5.4	1.20E-04	3.50E-02
UP_KEYWORDS	Collagen	RT	5	3.4	2.30E-03	3.70E-02
UP_KEYWORDS	Cell adhesion	RT	10	6.8	3.10E-03	4.50E-02
INTERPRO	EGF-like calcium-binding	RT	7	4.7	2.60E-04	4.90E-02

Supplementary table 1. Pathway analysis of C3H10T1/2 cells following miR-324-5p transfection. DAVID pathway analysis of the 200 most repressed transcripts following miR-324-5p transfection in Ihh stimulated C3H10T1/2 (identified by micro array), following miR-324-5p transfection.

	Species	Gene	Forward	Reverse	Notes
3'UTR generation					
	Human	GLI1	GCACTAGTAAGAGTAGGGAATCTCATCCATCACAGATCG	GCCAAGCTTCGTATGCAGTTCCTTTATTATCAGGAAACAGTGT	traditional
	Human	SMO	GCACTAGTGCCTGCAGAGCAGGACCTGGGACAGG	GCCAAGCTTAAAAACCTTTTATTGACTGTATTTCTTCTCC	traditional
	Human	GPC1	AAAGCTGCGCACTAGTGACAGAGGCCAAGGACTGAC	ATCCTTTATTAAGCTTCAAGGACTCAAAAGAACACACG	infusion cloning
	Mouse	Gli1	GGCACTAGTAAGGGTAAGGAACCCCAAGCAGATGGTATTT	GGCAAGCTTCAGTATTCCTTTATTGTCAGGAAACTGTGTCATT	traditional
	Mouse	Smo	AAAGCTGCGCACTAGTGGTCCTAGGATGGGAAAGACA	ATCCTTTATTAAGCTTCAAAAATGTTTTATTTGCTGTTCA	infusion cloning
	Mouse	Gpc1	AAAGCTGCGCACTAGTGTCCCCAAAAGCCATGTAT	ATCCTTTATTAAGCTTAAAGCGGTAAGCAGCCTTTT	infusion cloning
3'UTR mutagenesis					
	Human	GLI1	CACAAGATGCCCCAGGctacGGAGGTATGGGCTGGG	CCCAGCCCATACCTCCgtagCCTGGGGCATCTTGTG	
	Human	SMO	CACCCATTTAGTGGGcctaGGGTCCTCTAGACTTG	CAAGTCTAGAGGACCCtaggCCCACTAAATGGGTG	
	Mouse	Gpc1-site1	AGGAAGCCTGCAAGGctacCCAGTATGTTGCTGTC	GACAGCAACATACTGGgtagCCTTGCAGGCTTCCT	
	Mouse	Gpc1-site2	TCACCTGGCCATGGGctacCTGGGTGGCTGGTGAA	TTCACCAGCCACCCAGgtagCCCATGGCCAGGTGA	
	Mouse	Gpc1-site3	TTCCAGGGCCTAGGGctacCTGAGTTGCTATATCC	GGATATAGCAACTCAGgtagCCCTAGGCCCTGGAA	
Real-time PCR					
	Mouse	Gli1	Applied Biosystems Assays-on-Demand™	Applied Biosystems Assays-on-Demand™	
	Mouse	Gpc1	ATTGCCGAAATGTGCTCAA	GGCCCCAGAACTTGTCAGT	roche probe #79
	Mouse	18s	Applied Biosystems Assays-on-Demand™	Applied Biosystems Assays-on-Demand™	
Gpc1 over expression construct					
	Mouse	Gpc1	GAGCTCAACCGCGGATATCTAGAATGGAACTCCGGACCCGAGGCT	CTGGAATTGGGCCCAAATCTAGATTACCGCCACCTGGGCCTGGCT	

Supplementary table 2. Primers used for 3’UTR luciferase reporter cloning, site directed mutagenesis of 3’UTR luciferase reporters and real-time PCR. Nucleotides to mutate seed binding site are shown in small case.
